# eEurope 2002: Quality Criteria for Health related Websites

**DOI:** 10.2196/jmir.4.3.e15

**Published:** 2002-11-29

**Affiliations:** ^1^Commission of the European CommunitiesBrusselsBelgium

**Keywords:** Internet/standards, Ethics, Professional, Social Control, Formal, Health Care Quality, Quality Assurance, Health Care/standards, Commerce/standards, Information Management/standards, Medical Informatics/standards, Quality control, Guidelines, Privacy, Informed Consent

## Abstract

**Background:**

A number of organisations have begun to provide specific tools for searching, rating, and grading this information, while others have set up codes of conduct by which site providers can attest to their high quality services. The aim of such tools is to assist individuals to sift through the mountains of information available so as to be better able to discern valid and reliable messages from those which are misleading or inaccurate.

**Objective:**

Recognising that European citizens are avid consumers of health related information on the internet and recognising that they are already using the types of rating system described above, the European Council at Feira on June 19-20 2000 supported an initiative within eEurope 2002 to develop a core set of Quality Criteria for Health Related Websites. The specific aim was to draw up a commonly agreed set of simple quality criteria on which Member States, as well as public and private bodies, may draw in the development of quality initiatives for health related websites. These criteria should be applied in addition to relevant Community law.

**Methods:**

A meeting was held during 2001 which drew together key players from Government departments, International Organisations, non-governmental organisations and industry, to explore current practices and experiments in this field. Some sixty invited participants from all the Member States, Norway, Switzerland, and the United States of America took part in the meeting of June 7-8, 2001: they included delegates from industrial, medical, and patient interest groups, delegates from Member States' governments, and key invited speakers from the field of health information ethics. These individuals, and many others, also took part in the web-based consultation which was open from august to November 2001.

**Results:**

The broad headings for quality criteria identified include Transparency and Honesty, Authority, Privacy and data protection, Updating of information, Accountability, Responsible partnering, Editorial policy, Accessibility, the latter includes attention to guidelines on physical accessibility as well as general findability, searchability, readability, usability, etc. A metadata labelling system may be used to make health data more findable. Such a system may also be used in conjunction with quality criteria to give higher ranking by search engines to those sites or pages labelled as complying with defined quality criteria.

**Conclusions:**

The set of quality criteria is based upon a broad consensus among specialists in this field, health authorities, and prospective users. It is now to be expected that national and regional health authorities, relevant professional associations, and private medical website owners will 1) implement the Quality Criteria for Health Related Websites in a manner appropriate to their website and consumers; 2) develop information campaigns to educate site developers and citizens about minimum quality standards for health related websites; 3) draw on the wide range of health information offered across the European Union and localise such information for the benefit of citizens (translation and cultural adaptation); 4) exchange information and experience at European level about how quality standards are being implemented.

## Introduction

Health related web sites are now amongst the most frequently accessed sites on the internet with current estimates indicating that there are now over 100,000 sites offering health related information [1]. As a result of the wealth of information available and its apparent popularity, a number of organisations have begun to provide specific tools for searching, rating, and grading this information, while others have set up codes of conduct by which site providers can attest to their high quality services. The aim of such tools is to assist individuals to sift through the mountains of information available so as to be better able to discern valid and reliable messages from those which are misleading or inaccurate.

Recognising that European citizens are avid consumers of health related information on the internet and recognising that they are already using the types of rating system described above, the European Council at Feira on June 19-20 2000 supported an initiative within eEurope 2002 to develop a core set of Quality Criteria for Health Related Websites.

Accordingly a series of meetings was held during 2001 which drew together key players from Government departments, International Organisations, non-governmental organisations and industry, to explore current practices and experiments in this field. Some sixty invited participants from all the Member States, Norway, Switzerland, and the United States of America took part in the kick-off meeting of June 7-8, 2001: they included delegates from industrial, medical, and patient interest groups, delegates from Member States' governments, and key invited speakers from the field of health information ethics. These individuals, and many others, also took part in the web-based consultation which was open from august to November 2001 [2].

The focus of the discussions was primarily on the reliability of health related websites as a potential vehicle for health related messages, rather than on the substance and content of the health messages themselves. The specific aim was **to draw up a commonly agreed set of simple quality criteria on which Member States, as well as public and private bodies, may draw in the development of quality initiatives for health related websites. These criteria should be applied in addition to relevant Community law** [3].

As a result of the meetings, as well as a web-based public consultation, a core set of quality criteria was established. The criteria may be used as a basis in the development of user guides, voluntary codes of conduct, trustmarks, accreditation systems, or any other initiative adopted by relevant parties, at European, national, regional or organisational level. By using a common set of criteria as a starting point, such initiatives can develop in a focused manner across the European Union.

The objectives for the criteria were defined as follows:

- The quality criteria should address issues of both supplier and user education: one document that simultaneously tells suppliers how to comply with key quality criteria and educates users as to what they ought to expect from a good health website;
- The quality criteria should address both passive information-giving sites as well as sites that allow for transactions between service or information providers and users (i.e. information, products and services).
- The quality criteria should facilitate compliance with EU Directives, other current guidelines, and technical standards relevant to this area.

It should be noted that the objective was not to develop a method for the implementation of such criteria at a European level. Although some actors in the field have called for an EU trustmark for health related websites which would operate in a way similar to the CE marking of certain goods [4,5], such initiatives are not within the ambit of the eEurope2002 action. They may, however, be considered within future eEurope action plans and other European programmes.

**Quality Criteria for Health Related Websites**

Developed in widespread consultation with representatives of private and public eHealth websites and information providers, other industrial representatives, public officials, and representatives of government departments, international organisations, and non-governmental organisations.

These criteria should be applied in addition to relevant Community law

Transparency and Honesty

- Transparency of provider of site - *including* name, physical address and electronic address of the person or organisation responsible for the site (see Article 5 and 6 Directive 2000/31/EC on Electronic Commerce).
- Transparency of purpose and objective of the site
- Target audience clearly defined (further detail on purpose, multiple audience could be defined at different levels).
- Transparency of all sources of *funding* for site (grants, sponsors, advertisers, non-profit, voluntary assistance).

Authority

- Clear statement of sources for all information provided and date of publication of source.
- Name and *credentials* of all human/institutional providers of information put up on the site, including dates at which credentials were received.

Privacy and data protection

- Privacy and data protection policy and system for the processing of personal data, including processing invisible to users, to be clearly defined in accordance with community Data Protection legislation (Directives 95/46/EC and 2002/58/EC).

Updating of information

- Clear and regular updating of the site, with date of up-date clearly displayed for each page and/or item as relevant. Regular checking of relevance of information.

Accountability

- *Accountability*- user feedback, and appropriate oversight responsibility (such as a named quality compliance officer for each site).
- Responsible partnering - all efforts should be made to ensure that partnering or linking to other websites is undertaken only with trustworthy individuals and organisations who themselves comply with relevant codes of good practice.
- Editorial policy - clear statement describing what procedure was used for selection of content.

Accessibility

- *Accessibility*- attention to guidelines on physical accessibility as well as general findability, searchability, readability, usability, etc.

Relevant Community Law is listed in reference 3. Terms *in italics* are further discussed in the Glossary of Terms.

It should also be noted that while this Communication is addressed to the Member States of the European Union and private or public bodies operating in those States, due consideration should be given to the global nature of information disseminated through websites. Accordingly bodies adopting measures to implement the criteria should be aware of the fact that their information will be accessed by many individuals of different nations and cultures. In particular, attention should be paid to the fact that the developing world is a keen consumer of health information and that culturally specific content should be clearly identifiable as such.

Textbox 1 sets out the resulting quality criteria; the ensuing text then explores some of the ways in which they may be implemented. This illustrates what could be done at national or regional level to promote high quality, accessible health related information to the European citizen. The table may easily be detached from the present text to form a simple reminder of the key Quality Criteria for Health related Websites.

## Tailoring the Criteria For Different Types of Health Related Content

The criteria set out above are designed to be applicable to the development and maintenance of a health related site irrespective of the type of information or audience to whom the information is targeted. However, one essential quality criterion is that a health-related web site should state clearly what is its target audience and that care should be taken to ensure that both the style and nature of the information, and its presentation, are appropriate for the chosen audience. A number of the respondents to the consultation on the draft criteria, which was conducted between August and October 2001 via the eEurope website, identified the need to address not only site development and maintenance, but also the specific quality issues particular to health related content [6].

When tailoring the content to a chosen audience, a number of factors should be borne in mind in addition to those set out above which should govern the construction of a site. These factors may be considered under the same broad headings as the general site development criteria:

### Transparency of Health Related Content

- Transparency of the health related objectives of the provider of the information, including the purpose and objective of content provision, should be clearly defined and stated.
- Where advice or information on particular conditions, lifestyles or medications is given, funding from producers of products thereby implicitly or explicitly endorsed should be transparent to the site user.
- Existing Community legislation already contains information and transparency requirements. For example Article 5 of Directive 2000/31/EC on electronic commerce concerns the general information to be provided by an Information Society Services provider; Article 6 of Directive 2000/31 which concerns additional information to be provided in the case of commercial communications which are part of or constitute an information society service and Article 10 of Directive 95/46/EC on the protection of individuals with regard to the processing of personal data and on the free movement of such data also applies.

### Authority of Health Related Content Providers

- Where a policy of using only accredited medical professionals to generate content is adopted, this should be clearly stated and adhered to.
- Where a mixed group of content providers is used, (medical professionals, journalists, personal testimony, etc) the category of content provider of each item should be clearly identifiable.
- Where scientific evidence is cited, the sources of such evidence should be easily identifiable to the user.
- Where a medicinal product is recommended, EU legislation on Medicinal Product advertising should be adhered to, and any documents authorised by a regulatory authority should be made available to the site user.
- Where advice is offered, the site provider should always include a reminder that internet based advice, whether personalised or not, cannot replace a face to face consultation with a healthcare practitioner.

### Privacy and data protection of Health Data

- Where any personal information is collected and further processed by the site user, including data processing invisible to the users, the requirements of Directive 95/46/EC on the protection of individuals with regard to the processing of personal data and on the free movement of such data, in particular article 8 on sensitive and health data, should be carefully assessed and full compliance assured.

### Updating of Health Related Information

- Where specific health related data are provided, the relevance of such content should be regularly verified.

### Accountability for Health Related Content

- Where specific health related user feedback is provided by the site, particularly where personalised medical advice is offered, every effort should be made to ensure that such advice is bona fide and that advisors are suitably qualified to offer advice.

### Accessibility in Health Related Content

- Where a particular type of audience is targeted (eg children), the presentation and content of information should be appropriate to the chosen target audience.
- The use of a metadata labelling system may be used to make health data more findable. Such a system may also be used in conjunction with quality criteria to give higher ranking by search engines to those sites or pages labelled as complying with defined quality criteria.
- Apply International or European standards, wherever possible, in order to facilitate notably the interoperability between different services and the cross-border provision of web based health services.

## Implementation of the Quality Criteria For Health Related Websites

### Issues for the European Community

The purpose of the eEurope 2002 action on Quality Criteria for health-related Websites was to encourage the adoption of a common set of basic quality criteria for such sites. The issue of whether and how these criteria might be implemented at European level was not within the terms of the action. The implicit assumption was that this was a matter to be addressed in Member States at national or regional level, making use of the wide range of private and not-for-profit organisations which are already operating systems for implementing quality criteria for health-related websites.

In view of the rapid increase in health-related websites in the European Union and the increase in the number of European Union citizens consulting such sites, it could be argued that there would be merit in the Community establishing its own system for implementing agreed quality criteria. Such a Community-sponsored system would however require considerable resources to set up and operate, and it is unclear that it would offer clear value added to the Member States. The Commission therefore considers that at the moment the difficulties inherent in a Community system would outweigh any possible advantages. Nevertheless, the issue of how and how effectively quality criteria are being implemented is of considerable significance at the European level. To ensure that European citizens have access to reliable health information on the Internet implies not only that there is a consensus on the necessary quality standards, but that those standards are satisfactorily implemented right across the European Union.

This does not mean that the same method of implementation should be used everywhere - indeed it must be doubtful that any particular mechanism would be appropriate in all circumstances and in all countries For example, in pharmaceuticals the Commission is considering ways of meeting the growing demand by patients to be able to access information directly about their medicines. The Commission has included proposals within the current review of EU pharmaceutical legislation, Review 2001, to take account of this growing demand. This has also been recently reinforced by the work of the High Level Group on Innovation and Provision of Medicines - G10 Medicines - which has covered this area in their reports.

However, whatever system adopted, there should be clarity about the mechanisms being used in the different Member States and the extent of the involvement of the national and regional health authorities. With the forthcoming enlargement of the European Union, this requirement for transparency becomes even greater.

### Some Examples of Methods of implementing Quality Criteria

#### Simple Codes of Conduct

A number of organisations have adopted an approach similar to that described in this Communication, of setting up meetings and consultations between experts in order to establish by consensus a set of quality criteria. The eHealth Code of Ethics adopted in May 2000 by the Internet Health Coalition [7] is perhaps the best known of such `codes of conduct'.

The object of this and other similar codes is to offer a process of self-assessment by health site providers. However, a basic code of good conduct, or set of quality criteria will form the basis of all the approaches described below.

The way in which such codes are implemented varies. Where the code is adopted by an umbrella organisation, such as the Pharmaceutical Group of the European Union, then the organisation itself seeks to ensure that all members comply with the code. In other cases a code has been adopted for the purposes of in-house application only, as in the case of the American Medical Association. Although other organisations may cite the code, and claim to use it, the organisation developing the code makes no attempt to ensure that other parties are in fact implementing it.

The costs of the code of conduct approach are generally rather low, requiring only an initial outlay on meetings to draw up the code. However, the benefit of such codes can also be rather limited given the absence of effective enforcement mechanisms.

A code of conduct which addresses issues concerning the protection of personal could in itself form a Code of Conduct relevant to a specific area of practice as foreseen in Article 27 of Directive 95/46/EC on Data Protection. Any such draft community code, and amendments or extensions to existing Community codes, may be submitted to the Working Party established by Article 29 of Directive 95/46/EC on data protection. Similarly, a Code of Conduct which addresses the particular issues of electronic commerce in the health domain may be drafted in accordance with the framework foreseen in Article 16.1 of the Directive 2000/31/EC on Electronic Commerce.

#### Self Applied Code of Conduct or Quality Label

A next step in the implementation of a code of conduct can be characterised as the self applied quality label. In such a case a third party organisation develops a code of conduct and allows those who undertake to abide by the terms of the code to display a label, seal or logo which certifies compliance with the code.

The oldest, and perhaps best known, of such labels is the Health on the Net Foundation (HON) [8] label whose eight point set of quality criteria is currently used by more than 3000 internet sites worldwide. A site provider wishing to use the HON label has to make a formal application and a commitment to strictly observe all the HON code principles. Compliant sites identify themselves by the HON code hyperlink (or "active") seal displayed at a prominent location. The seal is termed `active' because clicking on it links the user to the HON site. Conformity with the HON code principles is verified by the team of checkers at HON. HON cannot prevent dishonest operators from simply cutting and pasting the HON code seal onto their Web sites in a bid to enhance their credibility. It does, however, conduct random checks on subscribers to ensure they remain compliant with the HON code. By way of additional policing, the Internet community is invited to report misuse of the label.

The costs of this system of applied labelling are not very high, requiring a relatively small team to process applications for use, maintain random checks of sites displaying the label and respond to any reports of misuse. The benefits may be significant in drawing to the attention of users the importance of the criteria inherent in the label. However, the benefits must be weighed against the requirement of the users of the sites to understand the nature of the label, and perhaps more importantly, to care about its aims and objectives.

#### User Guidance Tools

A further application of the code of good conduct takes the form of a user guidance tool. In this case compliance with a code is demonstrated not by a label, but by a link to a guidance tool which invites the user to check for him or herself if a site and its contents comply with pre-set criteria.

A typical such tool is displayed by the site as a logo on which the user may click to reveal a series of questions with which to interrogate the site so as to assess whether the information offered is trustworthy. Such tools may be specific to a particular type of information, such as DISCERN [9] which provides a brief questionnaire through which users gain a valid and reliable way of assessing the quality of written information on treatment choices for a health problem. Other tools seek to give guidance on the trustworthiness of any health-related information. An example of this is NETSCORING [10], which uses a questionnaire of 49 criteria falling into eight categories: credibility, content, links, design, interactivity, quantitative aspects, ethics, and accessibility. Yet other tools are targeted at particular categories of internet users. For example, the QUICK [11] tool seeks to provide children with a step-by-step guide to assessing health related information on the internet.

While such tools are frequently adopted for the guidance of users by national health portals (such as National Health Service DIRECT in England and Wales), they may also be used as site development tools by authors and publishers of information since they define the standards which users are entitled to expect.

The financial costs of the user guide are low, often not extending beyond the initial development costs. However, the burden of the use of this kind of tool falls on the internet user, mostly because of the time it takes to apply, which reduces the incentive to use it.

#### Filtering tools

Where a guidance tool is provided by a third party to a user to apply for him or herself, a filtering tool is applied to provide a searchable database of filtered and accredited information. Such filtering tools are often based on the gateway approach to organising access to Internet. The fundamentals of this approach are that Internet resources are selected for their quality and relevance to a particular target audience. They are then reviewed and resource descriptions created, which are stored, generally with the associated metadata, and generally in a structured database. The consequence of this effort is to improve the recall, and especially the precision, of Internet searches for a particular group of users.

An example of this type of tool is found in the OMNI site (Organising Medical Networked Information) [12] which provides a gateway to evaluated, quality Internet resources in health and medicine, aimed at students, researchers, academics and practitioners in the health and medical sciences.

The costs of such a filtering tool are relatively high in that a team of trained experts must be employed to search for, abstract and classify information on the internet in order that it may be entered into the database. The benefits of such a tool, for the initiated user, are also high since it provides a valuable shortcut to individual searches of the internet using non-specific search engines.

#### Third Party Quality and Accreditation Labels

The most advanced, and also most costly, of the mechanisms available for implementing quality criteria for health related websites, is the third party accreditation system. A third party issues a label to certify the compliance of the site with the criteria of evaluation.

A range of implementations fall into this category, from lower cost intra-organisation bodies for quality certification, acting in a similar to the notified bodies used in CE marking, to high cost external independent assessors who perform audits and grant accreditation.

At present no third party accreditation bodies are fully operational in Europe, although two noteworthy pilots are running in MEDCERTAIN (a demonstration project of the European Union "Safer Internet Action Plan") and TNO QMIC, a pilot study of the Netherlands Organisation for Applied Scientific Research.

In the case of MEDCERTAIN [13] a series of levels of accreditation are envisaged, starting with a self-certification label in which the provider of the site uses the MEDCERTAIN metalabelling system which incorporates a machine read language to describe and evaluate health information on the Internet. These labels are then in turn used to place a given item of site correctly within a gateway system, such as the OMNI system described above. The next level envisaged by MEDCERTAIN is one in which non-medical experts personally check the site for compliance with the level 1 tagging and also against the agreed set of quality criteria. The highest level involves medical assessment of the content and a rating of the content by relevant healthcare professionals.

The QMIC [14] system, on the other hand, envisages a system similar to the ISO 9000:2000 standard. The QMIC system is based on a complex set of standards drawn up by the third party (TNO in this case) but implemented by the site provider through an internal `quality certification body' who is in turn regularly assessed by the third party organisation to ensure that it is performing its function of internal quality assurance properly. The site, once duly assessed by the internal notified body is then admitted to a portal maintained by the third party who undertakes to ensure that the sites linked into the portal are applying the internal quality assurance system with due care.

## Purposes of Implementation of Quality Criteria For Health Related Websites

The general purpose of any quality initiative, whatever method of implementation is chosen, must be the protection of the consumer. However, in some cases that general purpose may be best achieved through educating the user of the service while in other cases the provider of the service will be the target of the quality initiative. In order to assist in the selection of an appropriate implementation method, the targeted purposes of the various methods are examined in more detail below

### Educating Users

In their daily lives as consumers of information delivered via the traditional media, most people learn to use a wide range of assessment tools: judging the nature of the outlet providing the information (a general or specialist bookshop or a work exclusively available from the author), the look and feel of the publication as a whole (a magazine with several contributions or a one page pamphlet). In addition, most people know whom to contact for further information (librarian, bookshop assistant, publisher).

In the world of internet content, however, it is less evident what are the relevant indicators of quality. It is for this reason that quality marks and user guides have proliferated, namely to educate the consumer and to provide a recognisable "quality" label which site creators may use to promote their sites. Accordingly, for such codes to be effective it is highly important that the public are informed about the existence of the Codes through public education campaigns.

### Assisting searchers

The purpose of quality marks is not, however, simply to provide access to qualified information but also to assist the citizen in coping with the torrent of information which a search on a health related subject might produce: it has been said that "trying to get information form the internet is like drinking from a fire hose. You don't even know what the source of the water is" [15]. In order to try to manage the flow from the fire hydrant into a steady stream from a tap, some organisations have developed and applied tools for rating web sites in order that they may offer pre-selected and more easily searchable sources to their consumers (see for example OMNI or MEDCERTAIN).

### Educating Site Providers

The problem is not only with the torrent of information, but also with the behaviour of its purveyors. Whilst it may take considerable effort to find an outlet for unusual or extreme ideas in the traditional media, virtually anyone with a modicum of computer skills and very little money can create their own website. The objective of many of the code of conduct initiatives is therefore to educate both the providers and consumers of information about the processes and good practices that a website should be able to demonstrate.

In order to educate not only the provider, but also the consumer of information a further set of actors have developed a wide range of user assessment tools. Such tools are usually in the form of on-line check lists which ask the consumer to check off types of information as they find them: statement of aim, explicit statement of source of information, explicit date of information, etc. Some may be rather short (HON), some quite detailed (NETSCORING); some are aimed at specific markets (DISCERN - for treatment choices) and some aimed at children (QUICK) to mention but a few.

### Assuring Quality

Most of the organisations publishing and administering such codes operate on a simple selflabelling processes in which the site provider undertakes to follow the code and in return displays its "trustmark" relying on spot check and vigilant users to identify those who are not complying with the given code of conduct. While this may not be as effective as a fully policed trustmark system of the type we are used to seeing as regards, for example, electronic products, it nonetheless addresses a need in a reasonably effective manner.

### Conclusions

The eEurope initiative was launched by the European Commission on 8th December 1999, with the adoption of the Communication `eEurope - An Information Society for all (COM (1999), 687 final, of 8.12.1999)'.

The "eEurope 2002 Action Plan - An Information Society For All", was adopted by the Commission on 14th June 2000, and politically endorsed by the European Council in Feira (Portugal) on 19-20 June 2000. It detailed the policy actions which are required to meet these objectives by 2002.

The eEurope 2005 Action Plan (COM (2002) 263 final, of 28.5.2002), was adopted by the Commission on 28 May 2002 and politically supported by the European Council in Sevilla (Spain) on 21 - 22nd June 2002. It, notably, set the objective for Europe to have, by 2005, " *Modern online public services*". To achieve this objective, one of the proposed actions is to promote e-health services. It also commits the Commission to monitor " *actions taken by Member States to make health information as accessible as possible to citizens as well as initiatives to implement quality criteria for web sites*".

In this respect, the *e* Europe 2005 Action Plan affirms that " *it is critical that e-health content and services are developed efficiently, are available for all and health related web sites comply with established quality criteria*".

With respect to the enlargement of the European Union it should also be noted that the eEurope+ Action Plan, which was adopted by the accession States to mirror the eEurope 2002 Action Plan, includes similar action on quality criteria for health related websites.

Accordingly it will also be important to monitor the activities undertaken pursuant to that Action Plan.

This Communication sets the scene for the implementation of a core set of quality criteria in Member States for health related web sites, within the context of the relevant existing Community legislation (as listed in footnote 3) and in accordance with the requirements of that legislation. The set of quality criteria is based upon a broad consensus among specialists in this field, health authorities, and prospective users. It is now to be expected that national and regional health authorities, relevant professional associations, and private medical website owners will:

- implement the Quality Criteria for Health Related Websites in a manner appropriate to their website and consumers.
- develop information campaigns to educate site developers and citizens about minimum quality standards for health related websites.
- draw on the wide range of health information offered across the European Union and localise such information for the benefit of citizens (translation and cultural adaptation).
- exchange information and experience at European level about how quality standards are being implemented.

Finally, within the context of the Information Society activities and as part of the implementation of the European Union public health programme, consideration will be given to the possibilities of developing and operating a joint action, with the plans drawn up under eEurope, to improve availability to the general public on the Internet of information on health matters, and considering the possibilities for establishing a system of recognizable Community seals of approval for Internet sites.

## Appendix 1

### Contributors To Workshop and Consultation On Quality Criteria For Health Related Websites

Representatives from Member State Government Departments, Regional Representations and EU Permanent Representations

- Bundesministerium für soziale Sicherheit und Generationen,A
- Ministry of Health, IT
- Ministry of Social Affairs, Public Health and Environment,BE
- Direction de la Santé, LU
- Permanent Representation of Germany, BE
- Norwegian Board of Health, Nor
- Ministry of Social Affairs, Public Health and Environment,BE
- Ministério de Saúde - Instituto de Gestaõ Informática eFinanceira da Saúde, PT
- Wales European Centre, BE
- Systems Unit - Department of Health and Children, IE
- Permanent Representation of Greece, BE
- Ministério de Saúde - Secretaria Geral de Saúde, PT
- Permanent Representation of Denmark, BE
- Ministry of Health, Welfare and Sport, NL
- Ministry of Health, DK
- Ministry of Health and Social Affairs, Sw
- Ministry of Social Affairs and Health, Fin
- National Board of Health and Welfare, SW
- Ministry of Solidarity and Employment, FR
- Department of Health, UK

Representatives from Industry and Industry Interest Groups

- AVENTIS, BE
- Cap Gemini Ernst & Young Belgium N.V./S.A., BE
- K.E.L., BE
- Globalink, FR
- Adamson-BSMG Worldwide, BE
- Infomedica, SW
- Diagnostics Consultancy, NL
- Baxter SA, BE
- FARON, NL
- Association of British Healthcare Industries - ABHI, UK
- Iqmed - International Healthcare Consultants, DE
- Basil Strategies & IHC, FR
- European Medical Devices Organisation, BE

Representatives from Academia

- University of Keele - representing TEAC Health Project, UK
- De Montford University, UK
- Centre recherche Informatique et Droit, BE
- Nottingham University- representing OMNI / BIOME, UK
- University of Heidelberg - MedCERTAIN Project, DE
- University of Oxford, UK
- University of Coimbra / VA-IEETA, PT

Representatives from Non Governmental Organisations, International Organisations and Special Interest Groups

- Standing Committee of European Doctors, BE
- AFGIS (Agency for standards in Health IT), DE
- BEUC (Consumer Groups), BE
- Norwegian Centre for Telemedicine, Nor
- European Public Health Alliance, BE
- European Health Telematics Observatory (EHTO), PT
- European Network of Health Promotion Agencies, BE
- World Health Organization, CH
- European Consumers Organisation, BE
- Health On Net Foundation (HON), CH
- PGEU/GPUE (Pharmacists), BE
- National Patients Consumers Federation (NPCF), NL
- European Health Telematics Association (EHTEL), BE
- TNO Prevention and Health, NL
- CEN/ISSS (standards), BE
- Inspectorate for Health, NL
- Association Internationale de la Mutualité, BE
- British Medical Association, UK
- FINOHTA/STAKES (national research org), Fin
- Internet HealthCare Coalition, UK
- Deutsches Krebsforschungs Zentrum, DE
- American Accreditation HealthCare Commission (URAC), USA

## Appendix 2

### Glossary of Terms: Definitions and Guidance Notes On the Terms Used In the Quality Criteria

Accessibility

As well as ensuring that data are correct within the terms of the site providers' definitions, effort should be made to make the content of a website accessible to people with disabilities, including sensory impairments and learning difficulties. Guidelines for making websites and their content accessible to all users have been developed in the Communication: eEurope2002: "Accessibility of Public Websites and their Content" (COM (2001)529f in of 25 September 2001).

Accountability

Accountability for a website is defined as a system by which anamed person or persons have a duty to respond to the questions andissues raised by users in a reasonable time. In a small organisation this may be one person who simultaneously performs many other tasks. Easy to use tools for providing feedback to asite should be used wherever appropriate.

Credentials

Where information is provided by a person or organisation on the basis of profession, such as physician, nurse, midwife or otherhealth professional, the qualification and where and when it was obtained, should be made clearly visible on the site. Where possible, links to the organisation issuing the qualification should be provided.

Funding

The term as used in the Guidelines includes any financial, material or in-kind support provided by organisations or individuals towards the development or maintenance of the website.

Interoperability

Interoperability is defined under Directive 91/250/EC [16] (Whereas 12) as "functional interconnection and interaction" and is "the ability to exchange information and mutually to use the information which has been exchanged;" In relation to web-based health services it is the possibility for two or more systems to functionally interconnect and interact.

Personal data

The term is used in the Guidelines within in the terms of **Directive 95/46/EC on Data Protection** to mean any information relating to an identified or identifiable naturalperson. An identifiable person is one who can be identified, directly or indirectly, in particular by reference to an identification number or to one or more factors specific to his physical, physiological, mental, economic, cultural or socialidentity.

From the outline presented above, it is clear that personal data exchanged in the process of any eHealth interaction between a patient and healthcare provider or between healthcare providers must comply with the requirements of the data protection Directives.

Processing of personal data

The term is used in the Guidelines within the terms of Directive95/46/EC as "any operation or set of operations which is performed upon personal data, whether or not by automatic means, such as collection, recording, organisation, storage, adaptation oralteration, retrieval, consultation, use, disclosure by transmission, dissemination or otherwise making available, alignment or combination, blocking, erasure or destruction"
